# Sensitive droplet digital PCR method for detection of *TERT* promoter mutations in cell free DNA from patients with metastatic melanoma

**DOI:** 10.18632/oncotarget.20354

**Published:** 2017-08-18

**Authors:** Ashleigh C. McEvoy, Leslie Calapre, Michelle R. Pereira, Tindaro Giardina, Cleo Robinson, Muhammad A. Khattak, Tarek M. Meniawy, Antonia L. Pritchard, Nicholas K. Hayward, Benhur Amanuel, Michael Millward, Melanie Ziman, Elin S. Gray

**Affiliations:** ^1^ School of Medical and Health Sciences, Edith Cowan University, Joondalup, Western Australia, Australia; ^2^ Anatomical Pathology, PathWest, QEII Medical Centre, Nedlands, Western Australia, Australia; ^3^ School of Medicine and Pharmacology, The University of Western Australia, Crawley, Western Australia, Australia; ^4^ School of Pathology and Laboratory Medicine, University of Western Australia, Crawley, Western Australia, Australia; ^5^ Department of Medical Oncology, Fiona Stanley Hospital, Murdoch, Western Australia, Australia; ^6^ Department of Medical Oncology, Sir Charles Gairdner Hospital, Nedlands, Western Australia, Australia; ^7^ QIMR Berghofer Medical Research Institute, Herston, Brisbane, QLD, Australia

**Keywords:** droplet digital PCR (ddPCR), *TERT*, melanoma, cancer

## Abstract

**Background:**

Currently mainly *BRAF* mutant circulating tumor DNA (ctDNA) is utilized to monitor patients with melanoma. *TERT* promoter mutations are common in various cancers and found in up to 70% of melanomas, including half of *BRAF* wild-type cases. Therefore, a sensitive method for detection of *TERT* promoter mutations would increase the number of patients that could be monitored through ctDNA analysis.

**Methods:**

A droplet digital PCR (ddPCR) assay was designed for the concurrent detection of chr5:1,295,228 C>T and chr5:1,295,250 C>T *TERT* promoter mutations. The assay was validated using 39 melanoma cell lines and 22 matched plasma and tumor samples. In addition, plasma samples from 56 metastatic melanoma patients and 56 healthy controls were tested for *TERT* promoter mutations.

**Results:**

The established ddPCR assay detected *TERT* promoter mutations with a lower limit of detection (LOD) of 0.17%. Total concordance was demonstrated between ddPCR and Sanger sequencing in all cell lines except one, which carried a second mutation within the probe binding-site. Concordance between matched plasma and tumor tissue was 68% (15/22), with a sensitivity of 53% (95% CI, 27%-79%) and a specificity of 100% (95% CI, 59%-100%). A significantly longer PFS (p=0.028) was evident in ctDNA negative patients. Importantly, our *TERT* promoter mutations ddPCR assay allowed detection of ctDNA in 11 *BRAF* wild-type cases.

**Conclusions:**

The *TERT* promoter mutation ddPCR assay offers a sensitive test for molecular analysis of melanoma tumors and ctDNA, with the potential to be applied to other cancers.

## INTRODUCTION

Telomerase reverse transcriptase (*TERT*) encodes the catalytic subunit of telomerase, a ribonucleoprotein responsible for maintaining telomere length of chromosomes which play an integral role in cell immortality. Using linkage analysis and high-throughput sequencing, Horn et al. [1] reported somatic mutations in 74% of metastatic melanoma human cell lines, 85% of metastatic melanoma tumor tissues and 33% of primary melanomas. These mutations are the result of a cytidine to thymidine transition in the promoter of the *TERT* gene, at chromosome 5, 1,295,228 C>T and 1,295,250 C>T, hereafter termed C228T and C250T. These mutations create a putative consensus ETS (E26 transformation-specific) /ternary complex factor binding motif (GGAA/T), which is associated with an increase in *TERT* expression [1, 2]. The presence of these mutations in cutaneous melanoma is associated with fast growing melanomas [3] and poor prognosis [4]. The co-existence of *TERT* promoter mutations with *BRAF* or *NRAS* mutations (in 55% of cases) is associated with poor disease-free and melanoma-specific survival [5]. *TERT* promoter mutations occur frequently in a number of other cancers: 80–90% of glioblastoma multiforme, 60% of hepatocellular carcinoma, 60% of bladder cancer, 70% of basal cell carcinoma, 50% of cutaneous squamous cell carcinoma and up to 30% of thyroid cancers [6-11] and are associated with aggressive disease in thyroid carcinoma [12], glioblastoma [13], neuroblastoma [14] and renal cell carcinoma [15]. Therefore, it is of significant clinical benefit to develop a non-invasive and sensitive test that determines the *TERT* promoter mutation status in cancer patients.

Molecular profiling of tumors to aid cancer prognosis and to identify actionable therapeutic targets has become routine practice in clinical oncology. Whilst tumor tissue samples are typically used for mutation analysis, access to the tumor for biopsy, and the quality and quantity of the sample may hinder detection, particularly when methods with limited sensitivity are employed. Commonly used methods include Sanger sequencing, melting curve analysis and pyrosequencing which have limits of sensitivity of 15%-20%, 10% and 5% respectively [16]. More recently, tumor related aberrations have been determined in plasma cell free DNA (cfDNA) [17-22]. This is referred to as “liquid biopsy”, a relatively non-invasive test that can be performed regularly and provides information from the sum of all tumors at any one time point. It is, therefore, a valuable biomarker for monitoring disease progression and response to therapy [19, 23].

Whilst a variety of methods have been used to detect mutations from circulating tumor DNA (ctDNA), Hindson et al., have shown droplet digital PCR (ddPCR) to be a highly sensitive platform, enabling absolute quantitation of mutant *BRAF* down to 0.001% allelic fraction [24]. Various studies have since shown the utility of testing mutant *BRAF* in plasma of melanoma patients using ddPCR [18, 23, 25-27]. In particular, our laboratory has demonstrated that ctDNA analysis allows tracking of patient response to therapy and resistance acquisition [23]. Given the high prevalence of the *TERT* promoter mutations C228T and C250T in cutaneous melanoma [5, 28], their addition to existing tests for detection of mutant *BRAF* and *NRAS* will allow monitoring of most melanoma patients using ddPCR. Furthermore, it has been shown that concurrence of mutations in the *TERT* promoter with *BRAF* or *NRAS* mutations predispose patients to fast growing and aggressive disease, thus detection of multiple mutations including mutant *TERT* could serve as a prognostic marker.

**Figure 1:**
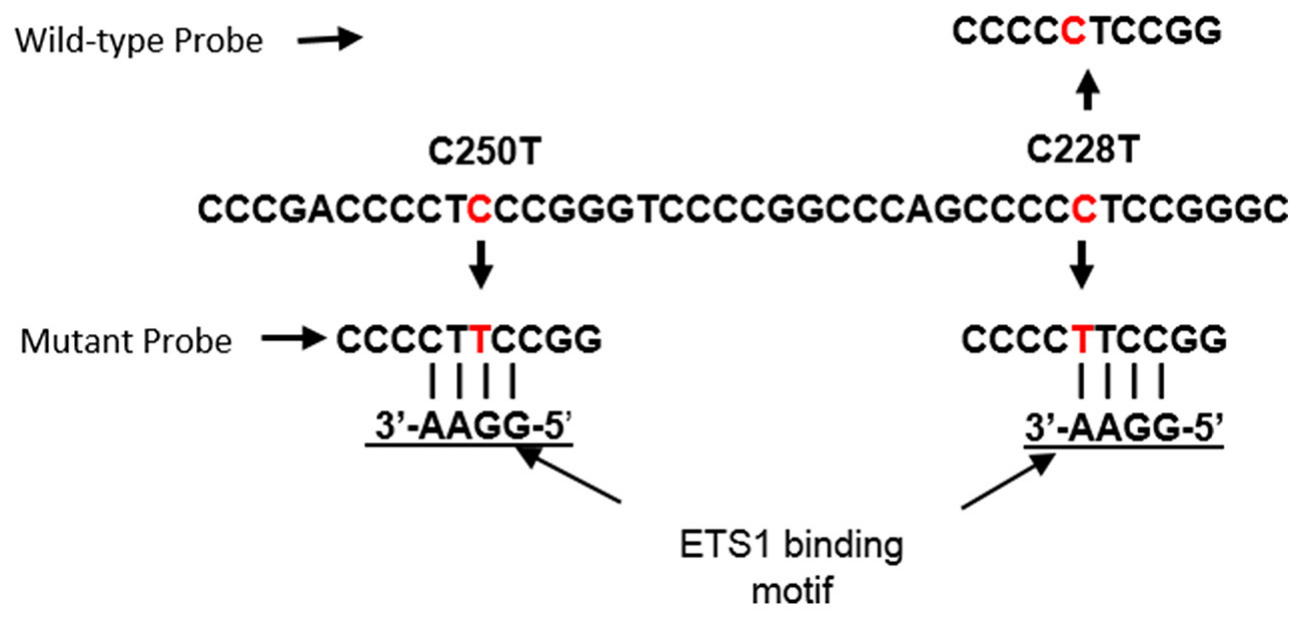
Location of ddPCR assay probes relative to ETS1 binding motifs generated by the C228T and C250T *TERT* promoter mutations Probes for the identification of wild-type and mutant sequences are indicated. Both mutant sites are detected by the same probe.

We report here on the development of a ddPCR probe based assay to simultaneously detect the *TERT* promoter mutations C250T and C228T. One probe binds the wild-type sequence overlapping position C228, while a second probe binds the mutant sequence resulting from C228T or C250T mutations, as both mutations reconstitute the putative ETS binding site (Figure 1). First, we tested the concordance of this assay for the detection of *TERT* promoter mutations in 39 melanoma cell lines relative to Sanger sequencing, and in 22 plasma samples relative to patient matched tumor tissue. We also determined the sensitivity and specificity of this assay for the detection of *TERT* promoter mutations using plasma derived cfDNA from 56 melanoma patients and 56 healthy controls.

## RESULTS

**Figure 2:**
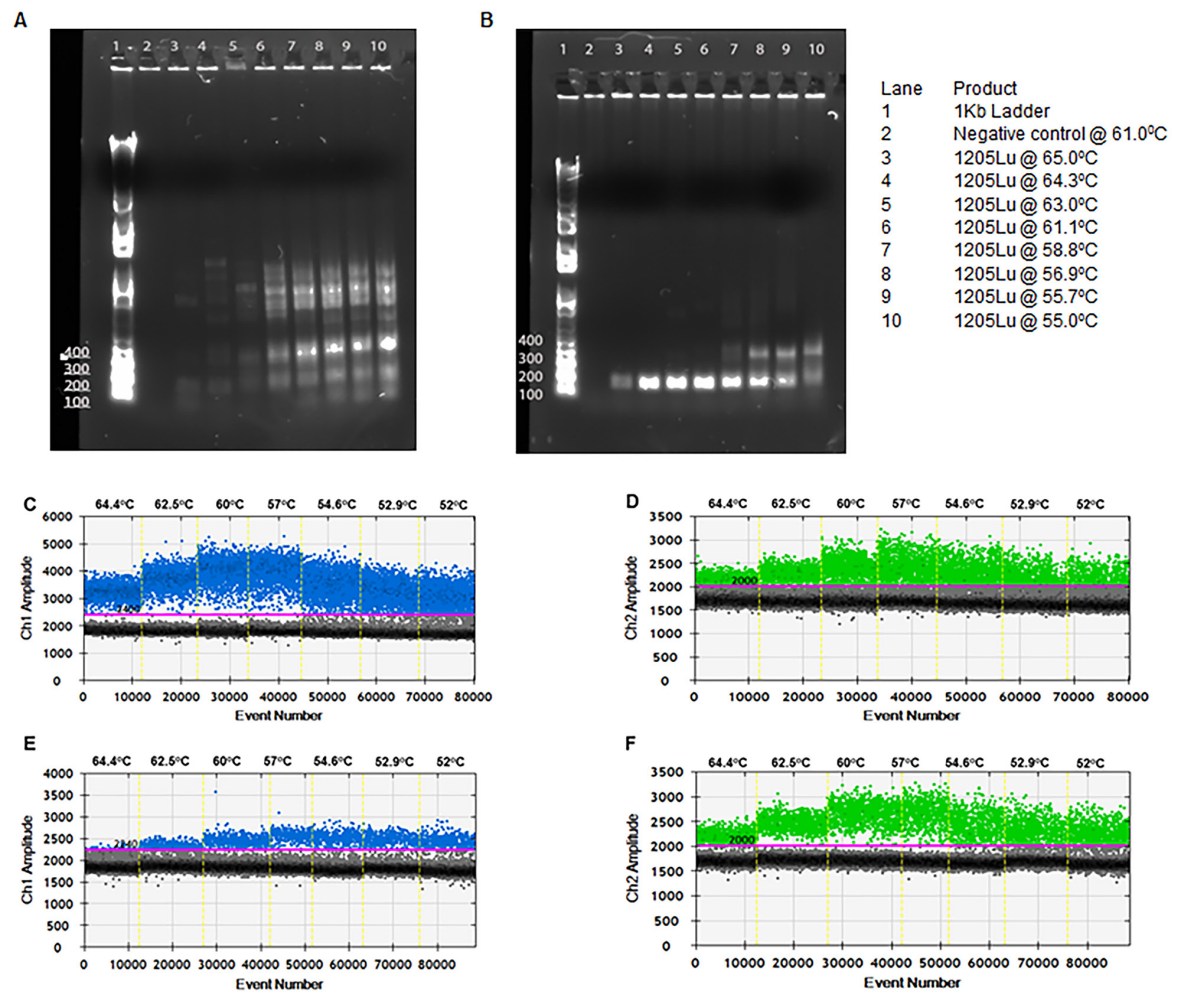
Optimization of ddPCR for detection of *TERT* promoter mutations PCR fragments from cell line 1205Lu amplified at varying temperatures without **(A)** and with **(B)** “Q solution”. gDNA of cell lines 1205Lu-C228T **(C** and **D)** and UACC62-C250T **(E** and **F)** were used as template for the *TERT* ddPCR at varying annealing temperatures. FAM signal from mutant probe binding to C228T (C) or C250T (E). HEX signal from binding of wild-type probe (D and F).

The designed primer sets were tested for amplification of the genomic region of interest by end-point PCR. Amplification conditions were optimized by testing a range of annealing temperatures (55-61°C). As shown in Figure 2A, the primers failed to amplify the required fragment in the absence of Q-solution (Qiagen). Optimal amplification was achieved in the presence of Q-solution between 61-64°C (Figure 2B). The PCR fragment obtained was subjected to Sanger sequencing to confirm its specificity.

Next, droplet digital PCRs were performed at a gradient of annealing temperatures from 52°C to 65°C for the detection of the C228T mutation in gDNA from 1205Lu cells (Figure 2C and 2D) and the C250T mutation in gDNA from UACC62 cells (Figure 2E and 2F). Optimal droplet segregation was observed at 57°C. Hereafter all ddPCR assays were performed with an annealing/extension temperature of 57°C.

**Figure 3:**
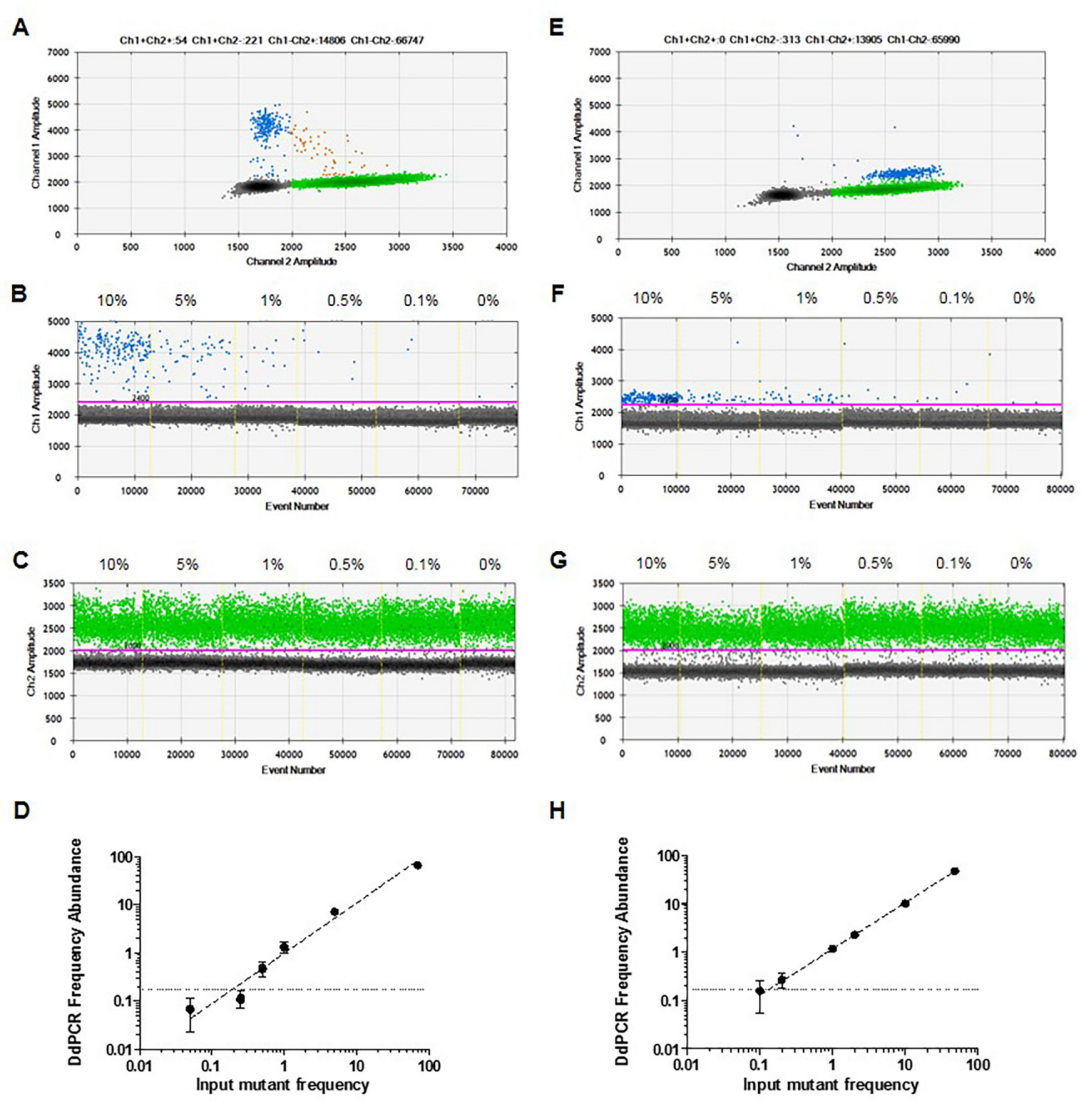
Detection of *TERT* promoter mutations in the presence of homologous wild-type DNA Serial dilutions of DNA from mutant cell lines 1205Lu – C228T **(A-D)** and UACC62 – C250T **(E-H)** were prepared in a constant background of wild-type human genomic DNA. 2D plots of ddPCR read out at 10% of mutant DNA (A and E). 1D plots indicating mutant (B and F) and wild-type (C and G) DNA detection. Analytical sensitivity (LOD) of the assay (D and H). Obtained frequency abundances and standard deviations were plotted versus expected mutant frequencies based on input. The LOD, defined as 2 SD over the mean frequency abundance obtained at 0% when only wild-type DNA was used as input, was indicated as dashed lines in both graphs.

To evaluate the quantitative linearity and the limit of detection (LOD) of the ddPCR assay, serial dilutions of mutant gDNA from cell lines 1205Lu (C228T mutant) and UACC62 (C250T mutant) were mixed in a background of wild-type human genomic DNA to achieve a final concentration of gDNA of 20 ng/μL (Figure 3), with each dilution tested in 8 replicates. At 0% mutant DNA, we identified that a maximum of 2 false positive droplets were observed in some of the 8 replicates, with an average of 0.068 ± 0.049%. Therefore, the lower LOD was defined at 0.17%, the percentage false positives detectable at two standard deviations over mean background [29].

To validate the assay, we tested 39 cell lines with known *TERT* promoter mutant or wild-type status (Table 1). We confirmed detection of the C228T and/or the C250T *TERT* promoter mutation in only those cell lines identified as positive for these two mutations, while those previously identified as wild-type showed no positivity for *TERT* DNA mutations by ddPCR. Cell lines that harbored an alternative *TERT* mutation other than C228T or C250T showed as wild-type in our assay. In addition, the C250T mutation was not detected in cell line C021, due to the presence of a C253T single nucleotide polymorphism in the probe binding site (Supplementary Figure 1). Simultaneous C250T and C253T mutations have been reported in 2% of melanoma cells lines [1].

**Table 1:** Validation of C228T and C250T *TERT* promoter mutation detection in melanoma cell lines

Cell line	Sanger sequencing	ddPCR
C024	wt	wt
C055	wt	wt
C092	wt	wt
C096	wt	wt
HGA	wt	wt
C022	C228T	C228T
C037	C228T	C228T
C058	C228T	C228T
D41	C228T	C228T
MM409	C228T	C228T
D22	C228T	C228T
MM473	C228T	C228T
A06	C228T^*a*^	C228T^*a*^
C076	C228T^*a*^	C228T^*a*^
MM455	C228T^*a*^	C228T^*a*^
1205Lu	C228T^*a*^	C228T^*a*^
A15	C250T	C250T
A14	C250T	C250T
C002	C250T	C250T
MM537	C250T	C250T
SKMEL13	C250T	C250T
MM386	C250T	C250T
D01	C250T	C250T
MM229	C250T^*a*^	C250T^*a*^
MM253	C250T^*a*^	C250T^*a*^
MM266	C250T^*a*^	C250T^*a*^
C001	C250T^*a*^	C250T^*a*^
C045	C250T^*a*^	C250T^*a*^
D40	C250T^*a*^	C250T^*a*^
UACC62	C250T^*a*^	C250T^*a*^
MM396	C227T/C228T	wt
A07	C227T/C228T	wt
C054	C227T/C228T	wt
C062	C227T/C228T	wt
C057	C241T/C242T	wt
C108	C241T/C242T	wt
D28	C241T/C242T	wt
SKMEL5	C241T/C242T	wt
C021	C250T^*b*^	wt

^a^Homozygous

^b^C021 carried an additional C253T polymorphism.

Tumor tissue samples from 22 stage IV (AJCC) metastatic melanoma patients were tested for C228T and C250T *TERT* promoter mutations by ddPCR using the *TERT* assay (Table 2). As reported in the literature [4, 28, 30], most tumor tissues tested harbored at least one of these mutations (68%, n=15); 11 harbored the C228T mutation and 4 harbored the C250T mutation. No tissue samples were found to contain both *TERT* promoter mutations.

**Table 2:** Detection of *TERT* promoter mutations in ctDNA and paired tumor tissue

	Tumor Tissue		
Plasma ctDNA	+	−	Total
**+**	8	0	8
**−**	7	7	14
**Total**	15	7	22

**Figure 4:**
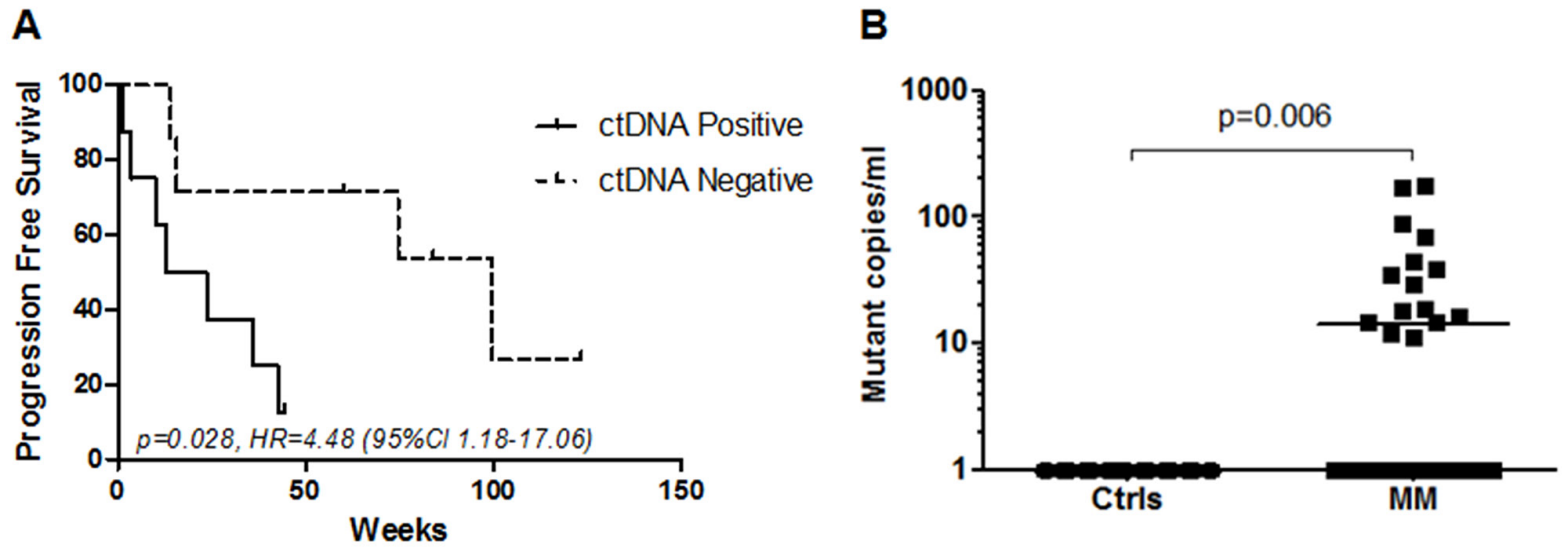
Detection of *TERT* promoter mutations in plasma **(A)** Kaplan-Meier plots of PFS probabilities of patients with detectable (n=8) and undetectable (n=7) ctDNA levels at baseline. Cox regression p-value, Hazard ratio (HR) and confidence interval (CI) are indicated. **(B)** Copies of mutant DNA per mL of plasma were significantly higher in metastatic melanoma patients (MM) (N=56) compared to healthy controls (Ctrls) (N=56). P=0.006, Mann-Whitney U-test.

Plasma derived cfDNA from these 22 patients were also tested for *TERT* promoter mutations. These plasma samples were collected from patients with active metastatic disease prior to any systemic therapeutic intervention. Overall, the concordance rate between tumor tissue and plasma testing was 68% (15/22). No patient was positive for a *TERT* promoter mutation in plasma and negative in its corresponding tumor tissue (100% specificity). Of 15 plasmas from patients with confirmed *TERT* promoter positive tumors, 8 were identified as positive for the same mutation, whereas 7 cases were positive in the tissue but negative in the plasma sample (Table 2). Thus, the sensitivity of our *TERT* C228T/C250T mutation detection in plasma was estimated as 53% (95% CI 27%-79%). In a cox regression analysis, patients with detectable ctDNA at baseline (n=8) had a significantly shorter progression free survival (PFS) compared to patients that had no detectable ctDNA (n=7) (*p*=0.028, Hazard ratio: 4.48 (CI, 1.18-17.06) (Figure 4A).

To further demonstrate the detection rate of *TERT* ctDNA in metastatic melanoma we tested 56 plasma samples from randomly selected stage IV (AJCC) melanoma patients (mean age 65 years, ranging from 35 to 85 years) with known *BRAF* but unknown *TERT* mutational status and compared this to 56 plasma samples from healthy individuals (mean age 51 years, ranging from 24 to 81 years). The *TERT* ddPCR assay detected a statistically significant difference in the copies of mutant *TERT* ctDNA in plasma from metastatic melanoma patients relative to those from healthy controls (p=0.006, Figure 4). We found *TERT* mutant DNA in 11 of 38 *BRAF* wild-type and in 4 of the 18 *BRAF* V600E/K patients. The number of *TERT* promoter copies per mL of plasma detected in the melanoma patient cohort varied from 11.2 to 176 copies per mL (Figure 4). No *TERT* promoter mutant DNA was detected in any of the 56 healthy control plasmas. Based on these results the assay specificity was estimated as 100% (95% CI 94%-100%).

## DISCUSSION

Here we describe and validate a method to detect the two most common *TERT* promoter mutations found in melanoma tumors using ddPCR. *TERT* promoter mutations occur in melanoma as frequently as [4], or more frequently [5] than *BRAF* mutations, and yet mainly *BRAF* mutant specific cfDNA is being used to monitor melanoma patients for response to therapy and disease progression [26]. The inclusion of *TERT* promoter mutations within ctDNA for monitoring would increase the number of patients for whom ctDNA could be used to determine disease status, particularly amongst *BRAF* and *NRAS* wild-type melanoma patients. This will enable large studies on the clinical utility of ctDNA monitoring to provide evidence of the efficacy of this marker for determining disease progression, to inform cessation of ineffective therapies [23, 27] and to guide alternative therapy.

Our assay allowed for detection of mutant *TERT* in biologically relevant samples, such as FFPE tumor DNA and plasma of metastatic melanoma patients at high specificity. Using cell line derived DNA, we optimized the assay to detect as little as 0.17% mutant *TERT* DNA in dilutions of wild-type DNA. This is significantly lower than limits of detection reported for other mutation detection platforms such as allele specific PCR at 1% [31] and pyrosequencing at 5%, melting curve analysis at 10% and Sanger sequencing at 20% [16]. While we and others have shown ddPCR to detect *BRAF* mutant fraction as low as 0.001% [24, 32], we were unable to achieve this sensitivity with the *TERT* assay developed here, possibly due to the highly GC rich area of the promoter region of this gene, resulting in background signal and limited segregation of positive and negative droplets. In fact, during the development of this assay, multiple primers, probes and amplification conditions were tested without success. The conditions detailed here, including the addition of LNA at the specific nucleotides and the use of Q-solution in the amplification mix, were indispensable for successful amplification.

We validated the assay in terms of accuracy and reliability by showing 97.4% concordance with the genotype of 39 melanoma cell lines. Of the cell lines analyzed that harbored either a C228T or C250T mutation, 14 were heterozygous and 9 homozygous. A major limitation of our assay is that it cannot detect other *TERT* promoter mutations and it can be affected by SNPs within the probe binding sites. This was apparent by the results obtained from 9 cell lines with known *TERT* promoter dinucleotide mutations C227T/C228T and C241T/C242T, which have been reported to exist in 5.2% and 10.4% of primary melanomas respectively [1]. Similarly, a negative result was reported for cell line CO12 which harbors a C253T SNP on the probe binding site. Further development of ddPCR assays to detect these other *TERT* promoter mutations [5] would ensure that a maximum number of patients could be monitored. In addition and given that SNPs in this region can also affect patient prognosis [5], germline sequence analysis should be performed complementary to the analysis of *TERT* promoter somatic mutations.

It is notable that all patients with *TERT* promoter mutations in plasma had corresponding mutations in matched tumor tissue and as such no false positive plasma samples were detected. High concordance between mutational profiles in plasma ctDNA and matched tumor tissue have been reported in several studies from patients with melanoma [18, 25, 26], breast cancer [20, 33, 34], non-small cell lung cancer [35, 36] and colorectal cancer [17, 20, 37]. In our study, 7 patients with *TERT* promoter positive tumors had no detectable *TERT* promoter mutations in matched plasma samples. This is similar to the findings by Lee et al, who detected ctDNA in 53% of patients prior to treatment initiation [38]. The lack of detectable ctDNA in a subset of patients may be explained by the pathophysiology of the tumor or its metastasis, as ctDNA concentration has been correlated with tumor size [38-40], metastatic spread or disease burden [25, 38, 41], tumor vascularization [42] and site of metastasis [20]. A retrospective analysis of PFS in this group of patients revealed a significant difference between patients with negative and positive ctDNA results. This further supports previous findings that low or undetectable level of ctDNA is a predictor of long term treatment benefit [18, 23, 25, 26, 38].

Previous studies have reported detection rates for *BRAF* V600E mutations in plasma of metastatic patients at 76 to 84.3% [25, 26] and for *BRAF* V600K at 81 to 89% [18, 26]. In other cancers, Bettegowda et al. [20] identified mutant ctDNA in 75% of patients with a variety of cancers including ovarian, breast, bladder, gastroesophageal and colorectal cancers. Considering our detection rates of *TERT* promoter mutations in ctDNA are lower (53%) than these reports, it would be necessary for this investigation to be conducted in a larger cohort controlling for tumor burden, metastatic sites and mutation variety. Nevertheless, our *TERT* promoter mutation assay allowed ctDNA detection in 11 of 38 *BRAF* wild-type tumors. Thus, our assay may facilitate ctDNA monitoring on *BRAF* wild-type cases, most of which will receive immunotherapy as a first line of treatment.

Nagore and colleagues [5] have shown that melanoma patients harboring these specific *TERT* promoter mutations, in combination with *BRAF*/*NRAS* mutations within their tumor tissue, have a significantly shorter disease free survival than patients without this combination. In fact, Li et al., have shown that *TERT* promoter mutations are key downstream targets of the RAS-ERK pathway for malignant progression of *BRAF* mutant melanomas [43]. Furthermore, Akincilar et al. [44] have shown that *TERT* transcription is driven by mediation of long-range chromatin interaction and enrichment of active histone marks through the recruitment of GABPA to mutant *TERT* promoters, specifically C228T and C250T. These authors have consequently suggested that inhibitors could be designed to hinder *TERT* transcription in cancer cells with these mutations. As such, routine genetic testing of melanoma patients for *TERT* promoter mutations in addition to mutant *BRAF* and *NRAS* would be clinically beneficial.

*TERT* promoter mutations have been identified in numerous other cancers such as thyroid, bladder, hepatocellular cancer and malignant glioblastoma [6-8]. Consequently, the assay described here may allow ctDNA monitoring in multiple other malignancies. However, the assay would require validation for each of these cancers.

In conclusion, we report on the development of a ddPCR assay for the detection of two common *TERT* promoter mutations in cell lines, tumor tissue and ctDNA. Our results suggest that the *TERT* ddPCR assay could prove useful as a companion diagnostic to predict treatment benefit and to monitor response in melanoma patients and could be extended to other malignancies.

## MATERIALS AND METHODS

### Ethics

This study was approved by the Human Ethics Committees at Edith Cowan University (No. 11543) and Sir Charles Gairdner Hospital (No.2013-246).

### Genomic DNA extraction

Genomic DNA (gDNA) with known *TERT* promoter mutations was obtained from melanoma cell lines 1205Lu (Wistar Institute) and UACC62 (National Cancer Institute) to be used as positive controls. In addition, gDNA was extracted from 39 melanoma cell lines from the QIMR Berghofer Medical Research Institute [45]. Wild-type gDNA was obtained from the white blood cell pellets collected from 4 mL whole blood from one healthy control. DNA was isolated using the QIAamp DNA Mini Kit (Qiagen, Australia) as per the manufacturer’s instructions. gDNA was eluted in AE buffer (Qiagen) and stored at 4°C until further processing.

### Plasma sample preparation

Blood samples were collected from American Joint Committee on Cancer (AJCC) stage IV melanoma patients, prior to initiation of any systemic therapy, into EDTA vacutainer tubes and stored at 4°C. Plasma was separated within 24 hours by centrifugation at 1600 g for 10 minutes, followed by a second centrifugation at 2000 g for 10 minutes, then stored at -80°C until extraction.

### DNA extraction from plasma

cfDNA was isolated from 5 mL of plasma from healthy donors and AJCC stage IV metastatic melanoma patients using the QIAamp Circulating Nucleic Acid Kit (Qiagen) as per the manufacturer’s instructions. cfDNA was eluted in 40 μl AVE buffer (Qiagen) and stored at -80°C until ctDNA quantification.

### DNA extraction from FFPE tissue

Following review and macrodissection by an experienced pathologist, genomic DNA (gDNA) was extracted from 10 x 5μm unstained sections of FFPE tissue using the QIAamp DNA mini kit (Qiagen) as per the manufacturer’s instructions. Only FFPE tissues stored at room temperature, for less than 7 years were used. The DNA concentration and purity was determined using the NanoDrop ND-1000 spectrophotometer (NanoDrop Technologies, Wilmington, DE) and Qubit 2.0 Fluorometer (Life Technologies, USA) instruments.

### PCR

The following primers were used to amplify a 163bp product incorporating both hotspot mutations (C228T and C250T) in the *TERT* promoter region: 5’-AGCGCTGCCTGAAACTCG -3’ (forward) and 5’-CCTGCCCCTTCACCTTCCAG -3’ (reverse). Primers were synthesized by GeneWorks (Thebarton, SA, Australia). For optimization of the PCR amplification of *TERT* promoter mutations, we first performed end point PCRs containing, 1 x ddPCR supermix (Bio-Rad), 900 nM of each primer and 50 ng of template gDNA, with and without 1 x Q solution (Qiagen). Amplifications were performed using the following cycling conditions: 1 cycle of 95°C for 15 minutes, 40 cycles of 95°C for 30 seconds and a range of temperatures from 55°C to 65°C for 30 seconds, followed by 68°C for 30 seconds and 1 cycle of 68°C for 10 minutes. PCR products of 163bp were detected by gel electrophoresis on a 1% agarose gel in Tris-acetate-EDTA (TAE) buffer containing SYBR® Safe DNA Gel Stain (Life Technologies).

### Droplet digital PCR

A probe was designed to detect both C228T and C250T mutation as both mutations result in the same sequencing string (Figure 1). Due to the short size of the probe, Locked Nucleic Acid (LNA) bases were introduced on the bases indicated with a “+” (TERT Mut:/56-FAM/CCC+C+T+T+CCGG/3IABkFQ/). A second probe was designed to recognize the C228 loci, also containing LNA bases, (TERT WT, /5HEX/CCCC+C+T+CCGG/3IABkFQ/). Probes were custom synthesized by Integrated DNA Technologies (IDT). Amplifications were performed in a 20 μL reaction containing 1 x ddPCR Supermix for Probes (No dUTP, Bio-Rad), 1x Q solution (Qiagen), 250 nM of each probe and 900 nM of each primer plus template.

Droplets were generated using the Automatic Droplet generator QX200 AutoDG (Bio-Rad). Amplifications were performed using the following cycling conditions: 1 cycle of 95°C (2.5C/s ramp) for 10 minutes, 40 cycles of 94°C (2.5C/s ramp) for 30 seconds and 57°C for 1 minute, followed by 1 cycle of 98°C (2.5C/s ramp) for 10 minutes. Annealing/extension temperature was optimized using temperature gradients from 52°C to 65°C. The sample was held at 4°C until further processing. Droplets were analyzed through a QX200 droplet reader (Bio-Rad). QuantaSoft analysis software (Bio-Rad) was used to acquire and analyze data.

To evaluate the LOD of our *TERT* ddPCR assay, gDNA from cell lines 1205Lu (C228T) or UACC62 (C250T) were serially diluted into normal human DNA obtained from white blood cells of healthy controls to achieve from 100% to 0% mutant alleles. Each dilution was tested in a series of eight repetitions all completed in one run.

Cell lines with known C228T and C250T *TERT* promoter mutations, as well as cell lines wild-type for both mutations (as determined by Sanger sequencing) were used to validate the assay. The reaction mix was prepared as above using 50 ng of gDNA as template.

For plasma ctDNA analysis, 5 μL of cfDNA (maximum template volume possible) was added per reaction irrespective of the cfDNA concentration. Each run included a non-template control, gDNA from a healthy control and gDNA from the cell lines containing the *TERT* mutations: 1205Lu (C228T) and UACC62 (C250T). Only samples with more than two positive droplets were considered positive. The number of mutated DNA copies per 20 μl reaction was extrapolated to calculate copies per mL using the following equation:

Copies/mL of plasma = C*EV/TV/PV.

PV = Volume of plasma used for cfDNA extraction (ml)

EV = Volume in which cfDNA was eluted (μl)

TV = Volume of cfDNA added to the PCR reaction (μl)

C = copies/20μl (data derived from QuantaSoft).

### Statistical analysis

Sensitivity and specificity of the assay was calculated using a contingency table analyzed using a Fisher’s exact test. Comparison between ctDNA concentrations in patient and control samples were performed using the non-parametric Mann-Whitney *U-*test. A Cox proportional hazards regression analysis was performed to examine association of ctDNA detection with PFS. Statistical analyses were performed using Statistical Package for Social Sciences for Window version 22 (SPSS, Chicago, IL) and plotted using GraphPad Prism version 5.

## SUPPLEMENTARY MATERIALS FIGURE


