# Supplementary material for: Sensitive droplet digital PCR method for detection of TERT promoter mutations in cell free DNA from patients with metastatic melanoma
Source: Oncotarget. 2017 Aug 18;8(45):78890–900. doi: 10.18632/oncotarget.20354 (PMC5668006; doi:10.18632/oncotarget.20354)
Supplement: Supplementary file 1 [file oncotarget-08-78890-s001.pdf]

Sensitive droplet digital PCR method for detection of *TERT* promoter mutations in cell free DNA from patients with metastatic melanoma

SUPPLEMENTARY MATERIALS

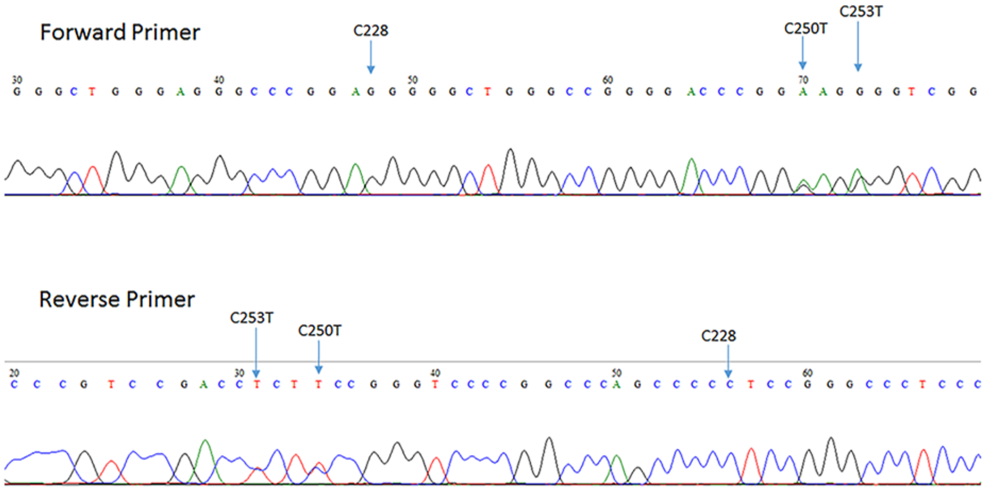

Supplementary Figure 1: Sanger sequence of *TERT* promoter of melanoma cell line C021. Electropherograms of forward and reverse sequence demonstrate the presence of a C253T mutation in addition to a C250T mutation in the *TERT* promoter.
